# National nutrition surveys in Europe: a review on the current status in the 53 countries of the WHO European region

**DOI:** 10.29219/fnr.v62.1362

**Published:** 2018-04-16

**Authors:** Holly L. Rippin, Jayne Hutchinson, Charlotte E. L. Evans, Jo Jewell, Joao J. Breda, Janet E. Cade

**Affiliations:** 1 Nutritional Epidemiology Group (NEG), School of Food Science and Nutrition, University of Leeds, Leeds, United Kingdom; 2 Division of Noncommunicable Diseases and Promoting Health through the Life-Course, World Health Organization Regional Office for Europe, UN City, Marmorvej 51, 21000 Copenhagen, Denmark

**Keywords:** national diet surveys, WHO European region, dietary assessment methodologies, scoping review – gaps, multi-criteria analysis, nutritional epidemiology

## Abstract

**Objectives:**

The objectives of this study were (1) to determine the coverage of national nutrition surveys in the 53 countries monitored by the World Health Organization (WHO) Regional Office for Europe and identify gaps in provision, (2) to describe relevant survey attributes and (3) to check whether energy and nutrients are reported with a view to providing information for evidence-based nutrition policy planning.

**Design:**

Dietary survey information was gathered using three methods: (1) direct email to survey authors and other relevant contacts, (2) systematic review of literature databases and (3) general web-based searches. Survey characteristics relating to time frame, sampling and dietary methodology and nutrients reported were tabled from all relevant surveys found since 1990.

**Setting:**

Fifty-three countries of the WHO Regional Office for Europe, which have need for an overview of dietary surveys across the life course.

**Subjects:**

European individuals (adults and children) in national diet surveys.

**Results:**

A total of 109 nationally representative dietary surveys undertaken post-1990 were found across 34 countries. Of these, 78 surveys from 33 countries were found post-2000, and of these, 48 surveys from 27 countries included children and 60 surveys from 30 countries included adults. No nationally representative surveys were found for 19 of 53 countries, mainly from Central and Eastern Europe. Multiple 24hr recall and food diaries were the most common dietary assessment methods. Only 22 countries reported energy and nutrient intakes from post-2000 surveys; macronutrients were more widely reported than micronutrients.

**Conclusions:**

Less than two-thirds of WHO Europe countries have nationally representative diet surveys, mainly collected post-2000. The main availability gaps lie in Central and Eastern European countries, where nutrition policies may therefore lack an appropriate evidence base. Dietary methodological differences may limit the scope for inter-country comparisons.

## Graphical Abstract: National diet surveys identified

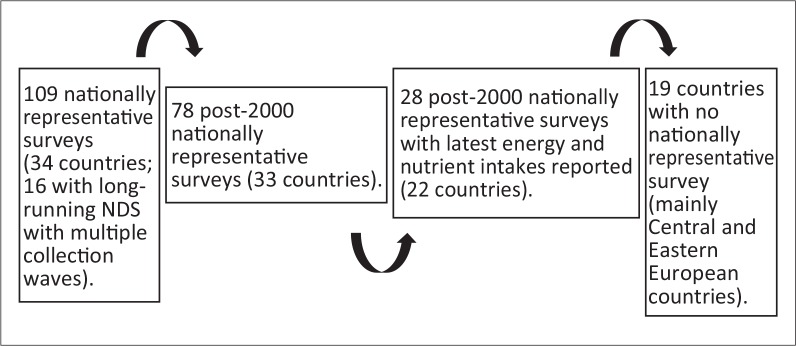



The World Health Organization (WHO) European Food and Nutrition Action Plan aims to ‘significantly reduce the burden of preventable diet-related noncommunicable diseases, obesity and all other forms of malnutrition still prevalent in the WHO European Region’ and improve diet and nutrition in the European population (1). An unhealthy diet is one of the four major behavioural risk factors for non-communicable diseases (NCDs) in all WHO regions (2), with the European region proportionately suffering the greatest NCD burden. Other risk factors include alcohol, tobacco misuse and physical inactivity (2). In Europe, the four most common NCDs account for 77% of disease and almost 86% premature mortality (1).

NCDs and related conditions, including overweight and obesity, have significant and growing economic and social costs (1), which traditional clinical approaches are increasingly unable to address (3). Mozaffarian et al. (3) call for a shift in emphasis from such pharmacological treatments to primary prevention through addressing lifestyle risk factors such as dietary patterns in order to reduce cardiovascular risk and NCD-associated problems.

Dietary surveys thus have an important role in assessing dietary patterns in the whole population. Nutrition and health surveys formed the main source of information for dietary risk factors and physical inactivity in a systematic analysis of disease risk in 21 regions worldwide across two decades (4). Such surveys can provide a means of monitoring trends, identifying areas of concern and inequality and evaluating policy impact, thereby ultimately contributing to the promotion of best practice across the region (1). The WHO European Food and Nutrition Action Plan (1) explicitly encourages member states to ‘strengthen and expand nationally representative diet and nutrition surveys’.

Many western European countries currently have established dietary surveys that assess food and nutrient intake. A global review of country-specific surveys from 1990 to 2010 only reported dietary fat and oil intake (5). A comprehensive, updated review of total nutrient and food intakes across different populations and subgroups in Europe is needed, the results of which could identify where in Europe there is a need to improve diets and whether inequalities exist. This paper makes the first step in this regard, establishing which countries have nationally representative dietary surveys and highlighting gaps in nutrition survey provision across Europe.

This review aims to identify which of the 53 countries in the WHO European region have conducted nationally representative dietary surveys of whole diets at an individual level and those that have not. It identifies key characteristics, centred on time frame, sampling and dietary methodology, of known surveys undertaken since 1990 for adults and children and aims to lay the foundations in establishing a clear picture of the current situation. Following this, future papers will examine energy and nutrient intakes in different population groups across Europe to better assess where both gaps in knowledge and dietary inadequacies lie. Information from dietary surveys can be used as a means for governments and health bodies to monitor and reduce the diet-related risk of NCDs and related conditions across Europe, thereby contributing to the goals set out in the WHO action plan.

## Methods

We used three key approaches to identifying national diet surveys: (1) contacting authors of surveys, (2) systematic literature review and (3) general web-based searches.

### Identifying authors of national diet surveys

We identified authors of national surveys within the WHO Europe remit using listed contact names and other information from two main reports of national dietary surveys (5, 6). If no response was obtained from those authors, Internet searches of nutrition organisations by country and the survey titles listed in the review of 1990–2010 surveys (5) and the European Food Consumption Survey (6) were carried out to find other potentially useful contact details. For countries where this approach did not yield usable contact details, Internet searches using various search terms were performed on organisations specialising in nutrition, including known government and public health agencies. WHO also provided contact details for some of those countries for which they had relevant associates. Contacts identified were asked to complete a questionnaire (Appendix 1) to provide information on nationally representative dietary surveys conducted at an individual level since 1990, including links or references to relevant reports.

### Systematic database search

For countries where no contact could be identified, systematic searches were undertaken across Web of Science, Medline and Scopus for nationally representative dietary surveys that collected data at an individual level from 1990 to June 2016. The following query terms were run without language restrictions: (survey* OR research* [TS]) AND (nutrition* OR diet* OR food* [TS]) AND (list of countries).

The title of each paper generated by the database searches was screened for relevance according to the criteria in Table 1; those that are not relevant were excluded. The remaining papers were screened by title and abstract, and full article where available, and their appropriateness for inclusion was checked by a second reviewer. Further surveys, related papers and nutrition expert contact names were gathered by general Internet searching to capture any recently released information, targeting known government and public health agencies using various search term combinations in order to maximise returns. Although there were no language restrictions in the initial search, the WHO Regional Office for Europe, Division of Noncommunicable Diseases and Promoting Health through the Life-Course, conducted an additional database search of papers in the Russian language as an extra check to maximise returns in the 12 Central and Eastern European countries where Russian is an official or widely spoken language (Armenia, Azerbaijan, Belarus, Georgia, Kazakhstan, Kyrgyzstan, Moldova, Russia, Tajikistan, Turkmenistan, Ukraine and Uzbekistan). However, no papers or reports that met the inclusion and exclusion criteria were found. The databases searched were PubMed, Web of Science and Google Scholar, using the search terms mentioned above, translated into Russian. Further searches with these terms were undertaken in three specific Russian language databases: Kazakh Academy of Nutrition; 1st Moscow Medical Academy named after Sechenov and Electronic Scientific Library in Russian.

**Table 1:** Survey inclusion and exclusion criteria

Included	Excluded
Surveys conducted at an individual level	Surveys collected at group, that is, household level
Nationally representative surveys	Non-nationally representative, regional only surveys
Results of surveys reported by published and unpublished reports, academic journals and websites	Surveys with data collected prior to 1990
Surveys that included individuals >2 years	Surveys with samples exclusively <2 years
Surveys based on whole diet rather than specific food groups	Surveys with incomplete food group coverage
	Surveys with small sample sizes (*n* < 200)

### Database extraction

Where long-running surveys had multiple collection waves, for example, the French INCA 1 and INCA 2 or UK NDNS 2000–1 and NDNS 2008–12, each collection wave was counted as a separate survey (see Table 2). Survey characteristics were extracted and tabled from the relevant publications, which were accessed in various forms, including summary reports, academic articles and completed questionnaires (see Table 2). The survey characteristics included the following: country name, survey name, year of survey (data collection), information source, sample size and age range, dietary methodology, nutrient composition database and reference. The availability of energy and selected nutrients from the latest surveys collected after 2000 are listed in Appendices 1 and 2.

**Table 2:** National diet surveys across the WHO Europe remit 1990–2016

Country*	Survey name	Survey year	Source **	Sample size	Sample age	Dietary methodology	Nutrient reference database†	Energy intake graphed Y/N‡	Reference
Albania	None found								
Andorra	Evaluation of the nutritional status of the Andorran population	2004–2005	6	900	12–75	24hr recall (×2 for 35% sample), FFQ. Face-to-face and phone interview.	CESNID. *Tablas de composición de alimentos*. Barcelona: Edicions Universitat de Barcelona-Centre d’Ensenyament Superior de Nutrició i Dietètica, 2002	Y	(32)
Armenia									
Austria*	Austrian nutrition report 2012 (OSES)	2010–2012	3	1,002	7–14; 18–80	3-day diary (consecutive) (children); 2×24hr recall (adults). Face-to-face and phone interview.	Analysis run with software ‘(nut.s) science’ based on Bundeslebensmittelschlüssel 3.01/Goldberg cut-offs for data cleaning.	Y	(33)
	Austrian study on nutritional status 2007	2007	4	2,472	7–100	Single dietary diary.		N	(34)
	Austrian study on nutritional status (ASNS)	1993–1997	5	2,065	19–95	24hr recall, diet history.		N	(35)
	Austrian study on nutritional status (ASNS)	1991–1994	5	2,173	6–18	7-day diary.		N	(36)
Azerbaijan	None found								
Belarus	None found								
Belgium*	Belgium national food consumption survey (BNFCS) 2014	2014–2015	2/3	3,146	3–64	2×24hr recall. Face-to-face electronic interview.	The NIMS Belgian Table of Food Composition (Nubel); Dutch NEVO	Y	(37, 38)
	Belgium national food consumption survey (BNFCS)	2004	3/4	3,245	15–100	2×24hr recall. Face-to-face interview. FFQ.		N	(39)
Bosnia and Herzegovina	None found								
Bulgaria	National survey on nutrition of infants and children under 5 and family child rearing, 2007	2007	3	1,723	0–5	2×24hr recall via mother (non-consecutive). Face-to-face interview with the mother.	FCTBL_BG (Food Composition Tables – Bulgaria)	Y	(40–42)
	National nutrition survey	2004	4	853	20–100	Single dietary diary.		N	
Croatia	None found								
Cyprus	A study of the dietary intake of Cypriot children and adolescents aged 6–18 years	2009–2010	3	1,414	6–18	3-day food record (consecutive inc 1 weekend). Self-completed.	USDA Nutrient Database for Standard Reference and Research	Y	(43)
Czech Republic	Individual food consumption study (SISP04)	2003–2004	2	2,590	4–90	2×24hr recall. Face-to-face interview.		N	(44, 45)
	Czech Post-MONICA Study	1997–1998	4	2,158	19–64	Single dietary diary.		N	
Denmark*	Danish national survey of diet and physical activity (DANSDA) 2011–2013	2011–2013	3	3,946	4–75	7-day diary (consecutive). Self-completed.	Danish Food Composition Databank	Y	(46)
	Danish national survey of diet and physical activity (DANSDA) 2003–2008	2003–2008	3	4,431	4–75	7-day diary (consecutive). Self-completed.		N	(47)
	Dietary Habits of Denmark 2000–2002	2000–2002	4	4,120	4–75	7-day diary.		N	(48)
	National dietary survey	1995	5	3,098	1–80	7-day diary		N	(49)
Estonia	National dietary survey	2014–15	2	4,906	4 months to 74 years	2×24hr recall (age >10); 2×24hr food diary (age <10); FFQ (age >2). Face-to-face electronic interview.		N	Not yet available.
	Nutrition and lifestyle in the Baltic Republics	1997	1/4	2,015	16–64	24hr recall + questionnaire		N	(50, 51)
Finland*	The National FINDIET 2012 survey	2012	3	1,708	25–74	48hr recall. Face-to-face interview.	Fineli 7 Food Composition Database	Y	(52)
	FINDIET 2007	2007	2/3/4	2,039	24–74	48hr recall. Face-to-face interview.		N	(53, 54)
	FINDIET 2002	2002	3	13,437	25–34, 35–44, 45–54, 55–64, 65–74	48hr recall. Face-to-face interview.		N	(55)
	FINDIET 1997	1997	5	3,152	25–74	24hr recall		N	(56)
	FINDIET1992	1992	4/5	1,861	25–64	3-day diary		N	(57)
France*	ESTEBAN	2015–16	2	3,617	Children 6–17 1,108; adults 18–74 2,509.	3×24hr recall		N	Not yet available.
	Enquête Nutri-Bébé 2013	2013	3	1,184	15 d–35 m	3-day weighed diary (non-consecutive). Face-to-face interview.		N	(58)
	Individual national food consumption survey (INCA2)	2006–2007	3	4,079	3–79	7-day diary (consecutive). Self-completed.	Food Composition Database of CIQUAL of Afssa.	Y	(59)
	Etude nationale nutrition sante (ENNS); National nutrition and health survey	2006–2007	2/4	4,780	Children 3–17 1,665; adults 18–74 3,115.	3×24hr recall (non-consecutive)		N	(60)
	Enquête Nutri-Bébé 2005	2005	3	706	1–36 m	3-day weighed diary (non-consecutive inc weekend). Face-to-face interview.		N	(61)
	Individual national food consumption survey (INCA)	1998–1999	5	1,018 1,985	3–14 15+	7-day diary.		N	(62)
	Enquête Nutri-Bébé 1997	1997	3	660	0–30 m	3-day weighed diary. Face-to-face interview.		N	(63)
	National food consumption survey (ASPCC)	1993–1994	5	1,500	2–85	7-day diary.		N	(64)
Georgia	None found								
Germany	German national nutrition survey (Nationale Verzehrstudie) II (NVSII)	2005–2007	2/4	15,371	14–80	DISHES diet history interview, 24hr recall, diet weighing diary (2×4 days). Face-to-face electronic interview.	Bundeslebensmittelschlüssel (BLS)	Y	(65, 66)
	Der Kinder- und Jugendgesundheitssurvey (KiGGS)	2003–2006	3	17,641	0–17	Questionnaire.		N	(67)
	German nutrition survey 1998	1997–1999	4/5	3,861	20–79	FFQ		N	(68)
Greece	HYDRIA – Greek national diet and health survey	2013–14	2	4,011	18+	2×24hr recall; food propensity questionnaire. Face-to-face interview.		N	(69, 70)
	Nutrient intakes of toddlers and pre-schoolers in Greece: The GENESIS study	2003–2004	3	2,374	1–5	3-day diary (includes nutrient data). Face-to-face interview.		N	(71)
Hungary*	Hungarian diet and nutritional status survey (OTÁP 2014)	2014	2	857	18–34, 35–64, 64+	3-day diary (non-consecutive). Self-completed.		N	Not yet available.
	Hungarian diet and nutritional status survey (OTÁP 2009)	2009	2	1,165	18–34, 35–64, 64+	3-day diary (non-consecutive), Self-completed.	Nutricomp.	N	(72)
	Hungarian dietary survey 2009	2009	3	3,077	19–30, 31–60, 60+	3-day diary (non-consecutive), FFQ, self-completed.	Új tápanyagtáblázat.	Y	(73, 74)
	3rd National Hungarian survey	2003	4	3,633	18–100	Multiple dietary diary.		N	(75)
	2nd National Hungarian survey	1992–1994	4/5	2,559	18–100	3×24hr recall + FFQ		N	(76)
Iceland*	The diet of Icelanders – a national dietary survey 2010–2011	2010–2011	2	1,312	18–80	2×24hr recall + FFQ. Telephone interview.	Icelandic Database of Food Ingredients (ÍSGEM); Public Health Institute for Raw Materials in the Icelandic Market.	Y	(77–79)
	The diet of Icelanders, dietary survey of the Icelandic nutrition council 2002	2002	4	1,118	15–80	Single dietary diary.		N	(80)
	Dietary survey of the Icelanders	1990	4/5	1,240	15–80	Diet history.		N	(81)
Ireland*	National pre-school nutrition survey	2010–2011	2	500	1–4	4-day weighed food diary (consecutive). Self-completed (by carer).	McCance and Widdowson’s The Composition of Foods 5&6 editions	Y	(82)
	National adult nutrition survey 2011 (NANS)	2008–2010	2	1,500	18–90	4-day semi-weighed food diary (consecutive). Self-completed.	McCance and Widdowson’s The Composition of Foods 5&6 editions	Y	(83, 84)
	Survey of lifestyle, attitudes and nutrition in Ireland (SLAN), 2007	2007	3/4	9,223	18+	FFQ. Face-to-face interview.		N	(85, 86)
	National teens’ food survey	2005–2006	2	441	13–17	7-day semi-weighed food diary (consecutive). Self-completed.	McCance and Widdowson’s The Composition of Foods 5&6 editions	Y	(87)
	National children’s food survey.	2003–2004	2	594	5–12	7-day weighed food diary (consecutive). Self-completed.	McCance and Widdowson’s The Composition of Foods 5&6 editions	Y	(88)
	SLAN 2002	2002	3	5,992	18+	Semi-quantitative FFQ.		N	
	SLAN 1998	1998	3	6,539	18+	Semi-quantitative FFQ.		N	
	North-South food consumption survey	1998	5	1,379	18–64	7-day diary. Self-completed.		N	(89)
	Irish national nutrition survey	1990	5	1,214	8–18+	Diet history.		N	(90)
Israel*	Mabat national health and nutrition survey of the Elderly (Zahav)	2005–2006	4	1,782	65–100	Single dietary diary.		N	
	Mabat first Israeli national health and nutrition survey	1999–2001	4	3,240	25–64	Single dietary diary.		N	
Italy	The third Italian national food consumption survey INRAN-SCAI 2005-2006	2005–2006	3	3,323	0.1–97.7	3-day diary (consecutive). Self-completed.	Banca Dati di Composizione degli Alimenti. INRAN-DIARIO 3.1	Y	(91)
	INN-CA 1994–1996	1994–1996	4/5	2,734	0–94	7-day weighed diary. Self-completed.		N	(92)
Kazakhstan	Nutritional and health status survey of the population in Kazakhstan	2008	6	3,526	15–59	2×24hr recall		N	
Kyrgyzstan	None found								
Latvia	National diet survey 2012–14	2012–2014	2	3,418	0–74	2×24hr recall (non-consecutive), FFQ, dietary diary		N	Results not yet available
	Latvian national food consumption survey 2007–2009	2008	2	1,949	7–64	2×24hr recall (non-consecutive), FFQ. Face-to-face interview.	Latvian National Food Composition Data Base 2009	Y	(93)
	Nutrition and lifestyle in the Baltic Republics	1997	1/4	2,299	19–64	24hr recall + questionnaire		N	(50, 51)
Lithuania	Study of actual nutrition and nutrition habits of Lithuanian adult population	2013–2014	2	2,513	19–75	24hr recall + questionnaire. Face-to-face interview,	EuroFIR Food Classification	Y	(94)
	Food consumption survey in adult Lithuanian population	2007	1/2	1,936	19–65	24hr recall.		N	(95, 96)
	Nutrition and lifestyle in the Baltic Republics	1997	1/4/5	2,094	20–65	24hr recall + questionnaire		N	(50, 51)
Luxembourg	None found								
Malta	None found								
Monaco	None found								
Montenegro	None found								
Netherlands*	Dutch national food consumption survey 2012–2016 (DNFCS 2012–16)	2012–2016	2	4,340	1–79	2×24hr recall and 1-day food diary (some age groups), FFQ.		N	Not yet available: (97)
	Dutch national food consumption survey 2007–2010 (DNFCS 2007–10)	2007–2010	2/3	3,819	7–69	2×24hr recall. Telephone (adults)/face-to-face (children) interview, FFQ.	Dutch Food Composition Database (NEVO)	Y	(98–100)
	Dutch national food consumption survey – young children (DNFCS 2008)	2005–2006	2	1,279	2–6	2-day diary (non-consecutive). Self-completed (by adult), FFQ.	Dutch Food Composition Database (NEVO)	Y	(101)
	Dutch national food consumption survey (DNFCS 2003)	2003	2/4	750	19–30	2×24hr recall (non-consecutive, telephone).		N	(102)
	Dutch national food consumption survey (DNFCS-3) 1997–1998	1997–1998	2/4/5	6,250	1–97	2-day diary.		N	(103)
	Dutch national food consumption survey (DNFCS-2) 1992	1992	2/4/5	6,218	1–92	2-day diary.		N	(103)
Norway*	UNGKOST 3	2015–2016	2	1,721	4–13	4-day online diary plus FFQ (consecutive). Self-completed via web.	The Norwegian Food Composition Tables	Y	(104, 105)
	Norwegian national diet survey NORKOST3	2010–2011	3	1,787	18–70	2×24hr recall and FFQ. Telephone interview.	The Norwegian Food Composition Tables	Y	(106)
	Sub-sample of NOWAC (component of EPIC)	2002	1	2,000 (female)	46–75	FFQ		N	(107)
	UNGKOST-2000	2000	3	394 815 1,009	4, 9 and 13	4-day diary, self-completed.		N	(108)
	Norwegian national dietary survey (NORKOST 1997)	1997	4/5	2,672	16–79	FFQ		N	(109)
	Norwegian national diet survey (NORKOST 1993–1994).	1993–1994	1/5	3,144	16–79	FFQ		N	(110)
	UNGKOST-1993	1993	5	1,705 1,564	13 18	FFQ		N	(111)
	Pilot study	1992	1	1,200	16–79	FFQ		N	(110)
Poland*	WOBASZ II study	2013–2014	3	6,170	20+	24hr recall and FFQ. Face-to-face interview.		N	(112)
	WOBASZ-national multicentre health survey	2003–2005	4	6,661	20–74	Single dietary diary		N	
	Sub-sample of the household food consumption and anthropometric survey	2000	4/5	4,200	1–100	24hr recall, face-to-face interview.		N	(113)
	Dietary habits and nutritional status of selected populations	1991–1994	5	1,126 2,193 4,945	11–14 18 20–65	24hr recall.		N	(114, 115)
Portugal	National food and physical activity survey (IAN-AF)	2015–2016	6	4,221	3 m–84 y	2×24hr recall (non-consecutive) and FPQ (electronic interview), 2-day food diary for children <10 years. Face-to-face electronic interview.	Portuguese Food Composition Table (INSA)	Y	(116, 117)
	Dietary calcium and body mass index in Portuguese children	2002–2003	3	4,511	7–9	24hr recall, face-to–face interview.		N	(118)
Republic of Moldova	None found								
Romania	National synthesis, 2006	2006	4	1,036	19–100	FFQ			
Russian federation*	The Russia longitudinal monitoring survey – higher school of economics (RLMS-HSE)	2011–2012	2/3	21,686	0–102	24hr recall.		N	(119)
	The Russia longitudinal monitoring survey – higher school of economics (RLMS-HSE)	1994, 1995, 1996, 1998, 2000, 2001, 2002, 2003, 2004, 2005	2	1994–11,295, 1995–10,632, 1996–10,448, 1998–10,663, 2000–10,969, 2001–12,100, 2002–12,489, 2003–12,634, 2004–12,639, 2005–12,228.	0–102	24hr recall.		N	(120)
San Marino	None found								
Serbia	None found								
Slovakia^‡‡^	Nutrient intake in the adult population of the Slovak Republic	1991–1994 & 1995–1999	1	4,018	19–80	24hr recall. Face-to-face interview.		N	(121, 122)
	Nutrient intake in children and adolescents in Slovakia	1991–1999	5	3,337 4,556	11–14 15–18	24hr recall and FFQ.		N	(122)
Slovenia	Dietary intake of macro – and micronutrients in Slovenian adolescents	2012	3	2,224	15–16	FFQ, self-completed.		N	(123)
	Dietary habits of the adult population Slovenia in health protection	2007–2008	2	1,193	18–65	2×24hr recall (non-consecutive), FFQ. Face-to-face interview,		N	(124)
Spain*	ENALIA 2 study	2014–2015	3	933 plus 157 pregnant women.	18–74	2×24hr recall, FFQ. Face-to-face electronic interview.		N	(125) Nutrient intake data not yet available.
	ENALIA study	2012–2014	3	1,780	6 m–17	2×1-day diary (<11 years); 2×24hr recall (11+); FFQ (all).		N	(126) Nutrient intake data not yet available.
	ANIBES study	2013	3	2,285	9–75	3-day diary + 24hr recall (consecutive). Face-to-face interview, telephone (interview), tablet and camera (self-report).	Tablas de Composición de Alimentos, 15ª ed	Y (children only)	(28–30)
	ENIDE study (Sobre datos de la Encuesta Nacionalde Ingesta Dietética)	2009–2010	3	3,000	18–24; 25–44; 45–64	3-day diary + 24hr recall (consecutive). Interview and self-completed.	Base de Datos Española de Composición de Alimentos – RedBEDCA	Y	(127–130)
	The Catalan nutrition survey (ENCAT 2002–2003)	2003	4	1,923	10–100	2×24hr recall (non-consecutive), face-to-face interview, FFQ.		N	
	EnKid study	1998–2000	3	3,534	2–24	24hr recall (×2 in 25% sample), face-to-face interview. FFQ.		N	(131, 132)
Sweden*	Riksmaten adolescents	2016–2017	2	?	11–12; 14–15; 17–19	2×24hr recall.		N	Data collection not yet completed.
	Riksmaten 2010–2011 Swedish adults dietary survey	2010–2011	3	1,797	18–80	4-day food diary (consecutive). Self-completed via web.	NFA Food Composition Database	Y	(133)
	Riksmaten-barn 2003 Swedish children’s dietary survey	2003	3	590, 889, 1,016	4 y, 8–9, 11–12	4-day food diary (consecutive), self-completed >4 years, by adult 4 years.		N	(134)
	Riksmaten 1997–1998	1997–1998	4/5	1,214	18–74	7-day diary.		N	(135)
Switzerland	MenuCH	2014–15	2					N	
	National nutrition survey Switzerland (NANUSS). Pilot for MenuCH.	2008–2009	2					N	
Tajikistan	None found								
The former Yugoslav Republic of Macedonia	First Macedonian food consumption survey	2015	6	504	16+	2×24hr recall. Interview.		N	Report not yet available.
Turkey	Turkey nutrition and health survey 2010 (TNHS)	2010	3	14,248	0–100	24hr recall, FFQ. Face-to-face interview.	BEBS Nutritional Information System Software; Turkish Food Composition Database	Y	(136, 137)
Turkmenistan	None found								
United Kingdom*	National diet and nutrition survey rolling programme Y5–6 (NDNS RP 2012–2014 )	2012–2014	3	2,546	1.5–94	4-day diary (consecutive). Self-completed.		N	(138)
	National diet and nutrition survey rolling programme (NDNS RP 2008–2012)	2008–2012	3	6,828	1.5–94	4-day diary (consecutive). Self-completed.	McCance and Widdowson’s The Composition of Foods integrated dataset	Y	(139)
	Low income diet and health survey (LIDNS)	2003–2005	4		2–100	4×24hr recall.		N	(140)
	NDNS 2000–2001 adults	2000–2001	4	1,724	19–64	7-day weighed dietary diaries.		N	(141)
	NDNS 1997 children	1997		1,701	4–18	7-day weighed dietary diaries.		N	(142)
	NDNS 1994–1995 65 years and over	1994–1995	4	1,275	65–100	Single dietary diary.		N	(143)
Ukraine	None found								
Uzbekistan	None found								

* Countries conducting long-running surveys comprising of multiple collection waves.

** 1 = database searches; 2 = email contacts; 3 = general Internet searches; 4 = Micha et al. (5); 5 = European Food Consumption Survey 2001 (6); 6) WHO Global Nutrition Policy Review 2017 extracted information.

† Information regarding nutrient composition databases has been added for those surveys for which energy and nutrient intakes were reported and graphed.

‡ Y = energy intakes were taken from the latest survey for which they were reported; N = energy and nutrient intakes were either not reported or were not extracted because intakes for that country were available in a later survey.

‡‡ The Slovakian surveys were not truly nationally representative, but were country-wide and designed to ‘recruit a diverse sample of subjects of different age categories and socio-economic background’ (121).

NB – The EFSA guidance for the standardised collection of national food consumption data was released in 2009.

## Results

### Data extracted

A total of 109 nationally representative surveys that obtained data on whole diets (rather than focusing only on certain foods) at an individual level since 1990 were found for 34 out of the 53 countries in the WHO office region. Table 2 shows the characteristics of these surveys and that the majority of countries with national dietary surveys (NDS) had conducted multiple surveys. Of the 34 countries with NDS, almost half (*n* = 16) had long-running surveys with waves conducted over various years; 10 of these also had stand-alone surveys (Table 2). Countries for which relevant survey characteristics were gathered are Andorra, Austria, Belgium, Bulgaria, Cyprus, Czech Republic, Denmark, Estonia, Finland, France, Germany, Greece, Hungary, Iceland, Ireland, Israel, Italy, Kazakhstan, Latvia, Lithuania, the Netherlands, Norway, Poland, Portugal, Romania, Russian Federation, Slovakia, Slovenia, Spain, Sweden, Switzerland, the former Yugoslav Republic of Macedonia, Turkey and the United Kingdom.

Of the 109 nationally representative surveys found, 78 were conducted since 2000, covering 33 countries – those listed previously, excluding Slovakia. Reports of energy and nutrient intakes were not found for each of these surveys. Only 28 surveys from 22 countries were found with post-2000 survey reports of energy and nutrient intakes.

**Fig. 1:**
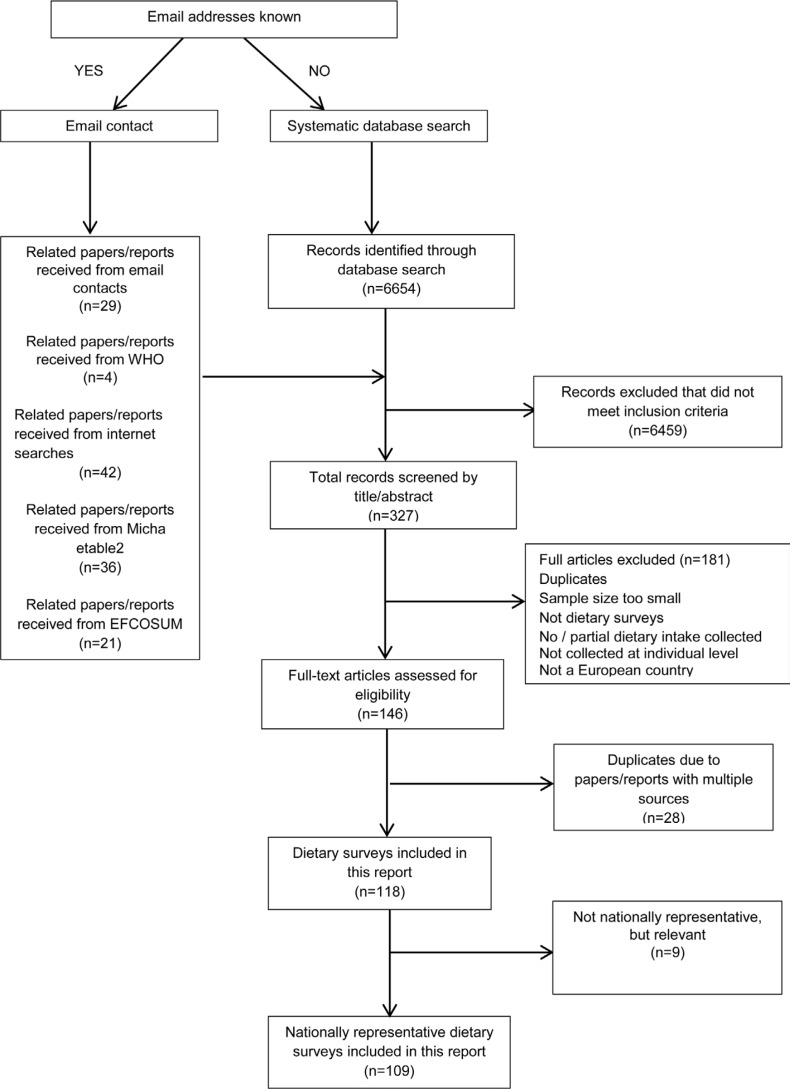
Screening and selection of national dietary surveys.

The majority of the surveys were found via Internet searches or emailing contacts gathered by the methods discussed. Current contact details were found for the following 30 countries: Austria, Belgium, Bulgaria, Croatia, Cyprus, Czech Republic, Denmark, Estonia, Finland, France, Germany, Greece, Hungary, Iceland, Ireland, Israel, Italy, Latvia, Lithuania, Malta, the Netherlands, Poland, Portugal, Romania, Russian Federation, Slovenia, Spain, Sweden, Switzerland and the United Kingdom. WHO provided details for Andorra, Kazakhstan and the former Yugoslav Republic of Macedonia. Contact details were not available for the following 20 countries: Albania, Armenia, Azerbaijan, Belarus, Bosnia and Herzegovina, Georgia, Kyrgyzstan, Luxembourg, Monaco, Montenegro, Republic of Moldova, San Marino, Serbia, Slovakia, Slovenia, Tajikistan, Turkey, Turkmenistan, Ukraine and Uzbekistan. For countries where no contact could be identified, the original systematic literature search returned 6,654 papers across the three databases, but only eight of these met the inclusion criteria. Of the 78 surveys undertaken since 2000, 30 papers or reports relating to them were acquired through email contacts, 4 from information extracted by WHO from the WHO Global Nutrition Policy Review 2017, 35 via Internet searching, 2 via the systematic literature search, 18 via the Micha review (5) and 1 from the EFCOSUM survey (6); 11 reports had multiple sources. See Fig. 1 for the full dietary survey screening and Table 2 for the characteristics of all dietary surveys conducted since 1990.

**Fig. 2:**
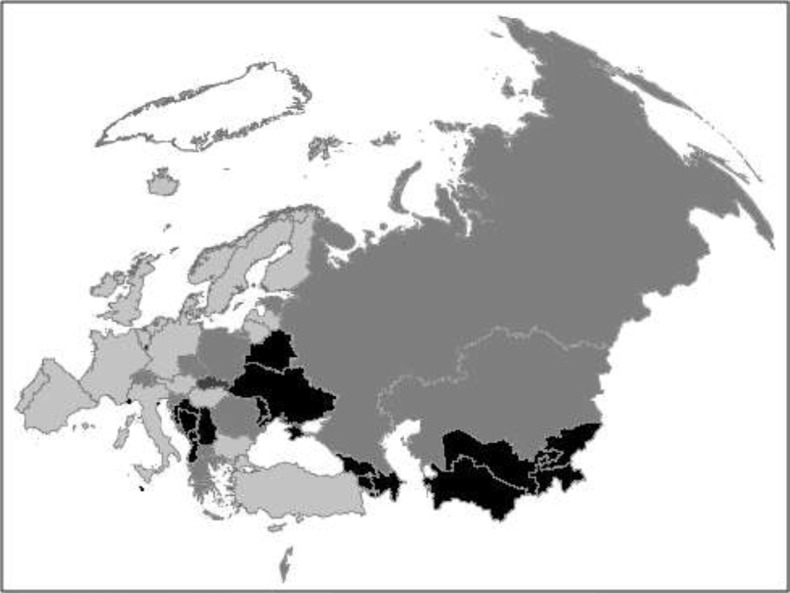
Map of national dietary survey provision by country. Light grey – Post-2000 survey plus nutrient intakes (28 surveys in 22 countries). Medium grey – Post-2000 survey (78 surveys in 33 countries). Medium-dark grey – Pre-2000 survey (3 surveys in 1 country). Dark grey – No survey (19 countries). White – countries not in the WHO Europe remit.

No nationally representative surveys were found by any method that collected dietary intake of whole diets at individual level for 19 European countries (see Table 3 and Fig. 2). Although one survey of children was found for Croatia, it was not nationally representative (7). In addition, no nationally representative surveys have been found for Slovakia that have been conducted since 2000, and none for Bulgaria and Czech Republic since 2005. In Western Europe, no surveys have been found for Italy or Israel conducted since 2006, or for Andorra since 2005.

**Table 3:** Level of nationally representative survey provision by country

Countries with no surveys	Countries with pre-2000 surveys only	Countries with post-2000 surveys without reports of energy and nutrient intakes	Countries with post-2000 survey plus energy and nutrient intakes
Albania	Slovakia	Czech Republic	Andorra
Armenia		Estonia	Austria
Azerbaijan		Greece	Belgium
Belarus		Israel	Bulgaria
Bosnia and Herzegovina		Kazakhstan	Cyprus
Croatia		Poland	Denmark
Georgia		Romania	Finland
Kyrgyzstan		Russian Federation	France
Luxembourg		Slovenia	Germany
Malta		Switzerland	Hungary
Monaco		The former Yugoslav Republic of Macedonia	Iceland
Montenegro			Ireland
Republic of Moldova			Italy
San Marino			Latvia
Serbia			Lithuania
Tajikistan			The Netherlands
Turkmenistan			Norway
Ukraine			Portugal
Uzbekistan			Spain
			Sweden
			Turkey
			United Kingdom

Of the 109 nationally representative surveys, 45 obtained dietary information on both adults and children, a further 41 surveys collected dietary information on adults aged 18+ only and 23 on children aged <18 only. For the 86 surveys that included adults, 60 across 30 countries were conducted since 2000. Of the 68 surveys that included children, 48 were conducted since 2000 and spanned 27 countries. Nationally representative surveys for children were missing in nine countries: Croatia, Finland, Hungary, Israel, Lithuania, Luxembourg, Romania, Slovakia and Switzerland. Further gaps were found for Andorran children aged <12 years; Bulgarian children aged above 5 years; Icelandic, Kazakh and Slovenian children aged <15 years; Macedonian children aged <16 years; Polish children post-2000 and Spanish micronutrient intake in children of all ages.

Non-nationally representative dietary surveys were found for eight countries (Croatia, Czech Republic, Germany, Greece, Iceland, Luxembourg, Russia and Switzerland), but because of our exclusion criteria they were not included in the list of nationally representative surveys in Table 2. Additionally, 16 studies conducted in Central and Eastern European countries were returned from the systematic literature search in English and 49 from the WHO Russian language database search and were not included in any tables; common reasons for rejection were no or partial dietary intake collected, data not collected at individual level, duplicate and sample size too small (<200). Eight countries completed the WHO STEPwise approach to noncommunicable disease risk factor surveillance (STEPS) adult survey (8–15). However, although these were nationally representative population-based surveys with large sample sizes, they were not included in this review because they only covered specific food groups, not whole diets, and as such did not meet our inclusion criteria.

### Dietary methodologies

The most common dietary assessment methodologies used across the 109 nationally representative surveys were the 24hr recall and food diary. Of these surveys, 45 used 24hr recall, 35 of which were surveys conducted since 2000 (Table 2). Of the 45 surveys using 24hr recall, the range of daily recalls was 1–4; 29 surveys used *multiple* 24hr recalls, 26 of which were conducted post-2000. Table 2 illustrates that where countries used both 24hr recall and food diaries, this was a combination of methodological changes in waves of long-running surveys, different surveys using different methodologies or both methods being employed within the same survey for different population groups, for example, adults and children. A 2×24hr recall is the method recommended by the European Food Safety Authority (EFSA) for adults’ NDS (16). Countries with surveys conducted post-2000 using multiple 24hr recall were Austria, Belgium, Bulgaria, Czech Republic, Estonia, Finland, France, Greece, Iceland, Kazakhstan, Latvia, the Netherlands, Norway, Portugal, Slovenia, Spain, Sweden, the former Yugoslav Republic of Macedonia and the United Kingdom. Spain calculated usual nutrient intake from 24hr recall and a 3-day dietary diary.

Food diaries were used as a primary method by 47 surveys, 33 of which were conducted post-2000. The range of diary days per survey was 1–7. Thirty-eight surveys used *multiple* day diaries as the primary method, and 26 of these were conducted post-2000 from the following countries: Austria, Cyprus, Denmark, France, Greece, Hungary, Ireland, Italy, the Netherlands, Norway, Sweden and the United Kingdom. The majority of these were performed over consecutive days. Weighed diaries were used as the sole method by some surveys in France, Ireland, Italy and the United Kingdom, but also as a primary method by one survey in Germany.

Food frequency questionnaires (FFQs) were used by 12 surveys, 5 of which were conducted post-2000 (Estonia, Ireland, Norway, Romania and Slovenia). FFQs were used by Ireland, Norway and Slovenia in pre-2000 surveys and as a supplementary, rather than primary, dietary assessment tool by other countries (Andorra, Belgium, Greece, Hungary, Iceland, Latvia, Lithuania, the Netherlands, Poland, Slovakia, Spain and Turkey).

Of the 28 surveys that reported energy and nutrient intakes (see Table 2 for older NDS approaches where available), 10 used interviews – these were primarily (*n* = 8) face-to-face rather than telephone-based, and 3 of these were electronic, for example, computer or tablet-based. Respondents self-completed in 11 surveys, which were all food diaries. Electronic resources were utilised in five surveys, just two of which were web-based. Five surveys used multiple approaches – these were mainly a combination of face-to-face and telephonic interviews with the exception of Spain, which used both interview forms plus a tablet and camera-photos.

### Energy and nutrient coverage

Of the 22 countries that had post-2000 nationally representative survey reports of energy and nutrient intakes, 20 countries reported data for adults and 16 countries for children. This was provided by 28 of the latest post-2000 surveys that reported energy and nutrient data for these countries; 13 surveys included both adults and children, 8 surveyed adults only and 7 sampled children only (3 being separate surveys of children in Ireland). Table 2 identifies these 28 surveys and illustrates their differing methodological approaches.

**Fig. 3:**
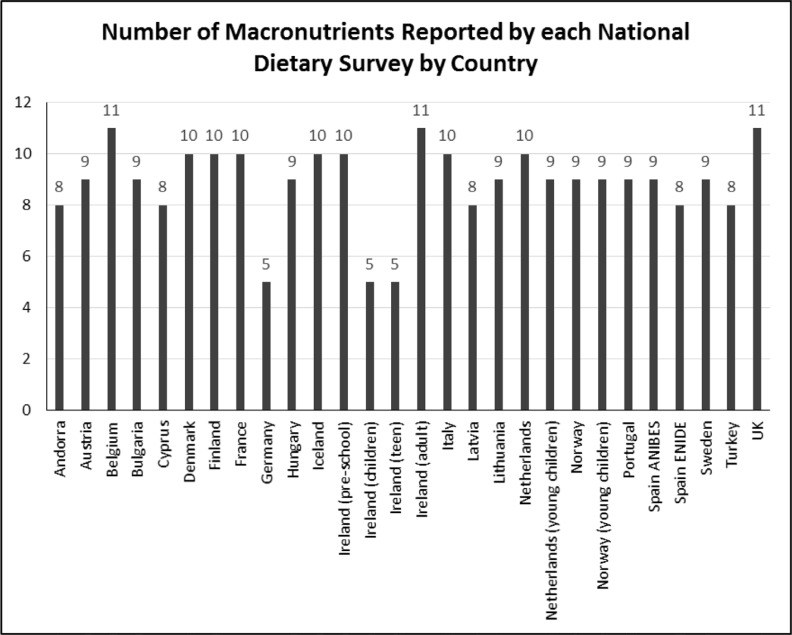
Number of macronutrients reported by each national dietary survey by country*: *Where 12 is the maximum potential number of selected macronutrients of interest being reported in NDS reports: energy, protein, carbohydrate, sugars, sucrose, starches, fibre, total fat, saturated fat, monounsaturated fatty acids (MUFA), polyunsaturated fatty acids (PUFA) and trans-fatty acids (TFA).

All 28 surveys included energy and also carbohydrate, fibre, fat and protein intakes. Most surveys (*n* = 25) included intake data on saturated fat (Germany and the Irish child and teen surveys did not): MUFAs (*n* = 25) (Germany, Irish child and teen surveys did not) and PUFAs (*n* = 24) (Germany, Irish child and teen surveys, and the Dutch DNFCS young children did not). See Appendix 2 and Fig. 3 for tabular and graphical summaries of the macronutrients included by each survey. The majority of surveys (*n* = 21) included intake levels of sugars in some form, either as total sugars or as added sugars or sucrose. However, Cyprus, Germany, the Irish child and teen surveys, Latvia, the Spanish ENIDE survey and Turkey included neither. Given current concerns about sugar consumption, this is an important gap. Few surveys (*n* = 6) included data on starch intakes and less than half (*n* = 9) included trans-fatty acid (TFA) intakes (see Appendix 2).

**Fig. 4:**
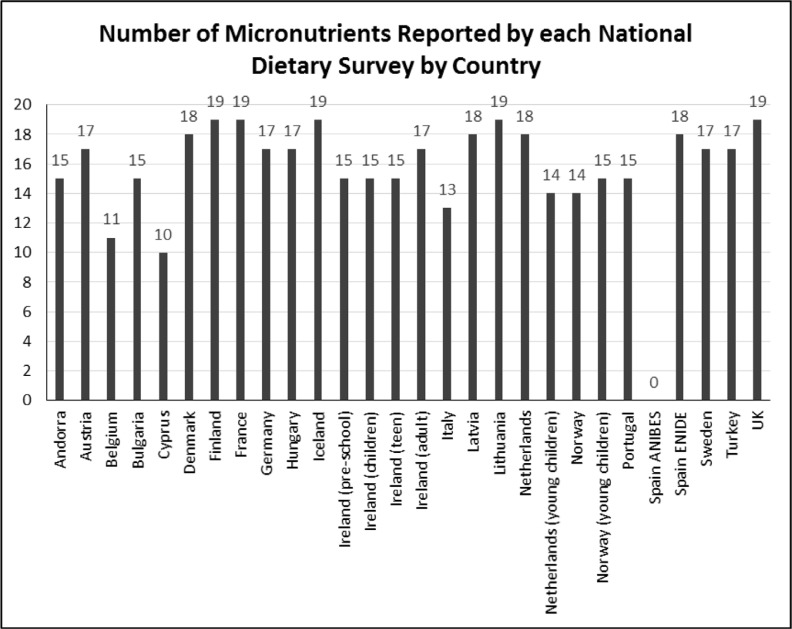
Number of micronutrients reported by each national dietary survey by country*: *Where 19 is the maximum potential number of selected micronutrients of interest being reported in NDS reports: folate (B9), niacin (B3), vitamin A, riboflavin (B2), thiamine (B1), vitamin B12 (biotin), vitamin B6 (pyridoxine), vitamin C, vitamin D, vitamin E, calcium, magnesium, potassium, sodium, iron, copper, iodine, selenium and zinc.

All surveys with the exception of the Spanish ANIBES study included some micronutrients of interest (see Appendix 3 and Fig. 4). However, none of the micronutrients investigated was reported by every survey. Vitamin A, riboflavin, thiamine, vitamin B6, vitamin B12, vitamin C, vitamin D, calcium, magnesium and iron were reported by 26 or more surveys. Copper (13), iodine (13), selenium (11) and fluoride (1 – not tabled) were reported by fewer than half the surveys.

## Discussion

### Data collection

This report details the initial findings of a review into dietary surveys across the 53 countries within the WHO Europe remit (17). Nationally representative surveys which collected data on whole diets at individual level since 1990 were found for only 64% of countries, the main gaps clearly lying in 17 countries in the Central and Eastern European region of the WHO Europe remit. Although eight countries without NDS had recently completed a comprehensive WHO STEPS survey, including questions on fruit and vegetable intake, salt consumption and use of fats and oils in cooking and eating, the survey does not address whole diets and only included adults; therefore, this represents a knowledge gap. However, non-nationally representative surveys were found in two countries that had no other NDS, which demonstrates that although some countries have no nationally representative surveys, other initiatives are in place and the expertise and fieldwork experience needed to conduct NDS may be present. All Western European countries had published survey information after 2000. Of countries with NDS, 16 conducted long-running surveys with multiple collection waves, which could generate important information for trends analysis. Fewer surveys were available that measured diet in children than adults; again gaps were primarily in Central and Eastern European countries. This implies that nutrition policies in this region are based on limited data, which is of concern, as overweight and obesity have tripled in some of these countries since 1980 and NCD prevalence rates are reaching those of Western Europe (1).

Emailing nutrition experts and general Internet searches were the most successful data gathering methods. A major source for contacts and survey information was a global survey review from 1990 to 2010 (5). Few academic papers met the pre-set inclusion criteria in the systematic database search performed for countries – particularly Central and Eastern countries – with no surveys or contacts mentioned in previous reviews, which also minimises the risk of bias. A possible explanation is that survey results and characteristics may be published as government or other official reports rather than academic papers. However, we also undertook wider web-based searches, targeting known government and public health agencies using various search terms to account for this. Another reason is that dietary assessment in large-scale studies like national diet surveys is costly, due to the labour-intensive nature of study preparation and data collection, and therefore may not be undertaken by some countries (18). This could explain the disproportionate concentration of gaps in survey provision in Central and Eastern European countries, which tend to have lower national incomes (19). This highlights a need to clarify major barriers and work with countries to establish mechanisms to overcome these and subsequently to devise and implement NDS.

### Dietary methodologies of post-2000 surveys

The most common methods of collecting dietary intake used in the 78 post-2000 surveys were the 24hr recall and food diary, the majority of which were collected over multiple days. Although 24hr recalls are known for under-reporting (20), their increased use could reflect their advantage in being less onerous for respondents and potentially providing more consistent results across all age and sex groups compared with other methods (21). Retrospective dietary recalls can provide detailed information on eating patterns and exert less influence on food choice than food diaries (22), thereby generating a more accurate and realistic report on population nutrient intake. However, such short-term dietary assessment methods are associated with within-person errors and wider variation of intakes within the population, particularly when intakes of only 1 or 2 days are collected, the latter as recommended by EFSA (16). Although FFQs provide long-term assessment, they nevertheless can present inflated energy and nutrient intakes (21), which could explain why few post-2000 surveys used FFQs as the primary dietary assessment method.

Prospective weighed and non-weighed food diaries allow very detailed information to be gathered on multiple days (22) and are sometimes used to validate other methods using a small sub-sample, but have a high respondent burden and like the 24hr recall, are susceptible to under-reporting (23). Food dairies with weighed intake are particularly burdensome and prone to response bias and respondent fatigue (24) – most likely the reason why fewer studies used it as a primary assessment method and the United Kingdom moved from weighed intake to estimated intake.

Many studies used multiple tools to collect food intake. Of the 22 countries for which energy and nutrient intakes were reported, all surveys that collected dietary intake using more than one tool generated energy and nutrient intake data from a primary method and used the other method(s) as a means of validation and calibration. The exception was Spain, which was the only country that used a truly mixed methods approach. Food diaries and 24hr recalls do not provide insight into usual intakes, whereas FFQs are less accurate in estimating individuals’ absolute intakes; combining methods could help rectify these shortcomings (24). Spain, Belgium and the Netherlands estimated ‘usual’ intakes using the Statistical Program to Assess Dietary Exposure (SPADE), although the Dutch intakes presented by age group in this report reflect the average of actual intakes reported by individuals. Of the other countries employing FFQ as a supplementary method, Greece and Iceland also explicitly stated that this was used to estimate usual intake. This approach is designed to overcome within-person errors and wider intake variations when only 2 days of intake have been collected, although methodological limitations cannot be fully negated.

Of the 23 surveys that sampled children only, over half (*n* = 15) used some form of food diary. This could be because children are expected to remember less retrospectively, so prospective methods of capturing intake, although subject to under-reporting and the limitations mentioned above, are deemed preferable and more accurate. This also fits with EFSA guidance on the collection of national food consumption data, which recommends countries ‘…use the dietary record method for infants and children and the 24-hour recall method for adults’ (16). EFSA further recommend data be collected on two non-consecutive days and that they be supplemented with a food propensity questionnaire (16). It remains to be seen whether more countries will move towards non-consecutive diaries in future surveys; at present, the majority of multiple food diaries are conducted on consecutive days. More detailed methodological recommendations for NDS of children are available via the Pilot study for the Assessment of Nutrient intake and food Consumption Among Kids in Europe (PANCAKE) project (25).

Of the 28 surveys that reported energy and nutrient intakes, Austria, Estonia, Iceland and Norway moved to 2×24hr recall in the latest NDS, perhaps to comply with the latest EFSA guidance (16). The United Kingdom switched from a 7-day weighed to a 4-day estimated food diary, which is more likely a move to reduce respondent burden. Although methodological changes make comparisons problematic across survey waves, the move towards a common approach will ease comparisons between countries in the long term and should be actively encouraged in line with EFSA recommendations. Although this could be logistically and financially challenging, it would assist in making inter-country comparisons and identifying vulnerable groups, thereby enabling the effective targeting of policy resources.

#### Technology in national dietary surveys

Care is needed in any dietary assessment method to minimise measurement error. Many dietary assessment methods require highly skilled interviewers, which increases survey costs and presents a potential barrier to conducting NDS (24). Technology like computer-administered interviews and image-capture could help overcome this obstacle and also promote standardised practices. The European Prospective Investigation into Cancer and Nutrition (EPIC)-Soft software package developed by the EPIC Study provided uniform templates for various aspects of NDS including conducting 24hr recall, which has since been modified by the European Food Consumption Validation (EFCOVAL) Study and renamed ‘Globodiet’. It aimed for Europe-wide use, but is limited by the need for professionals to be trained in its use (26).

At present, none of the surveys identified used mobile technologies to collect dietary information; although Belgian, German and Portuguese surveys employed electronic interviews, the Spanish ANIBES used tablets and the Norwegian Ungkost3 and Swedish Riksmaten used a web-based food diary. This current lack of use may be due to the lack of validation or differential usability across population groups. However, web-based dietary assessments with self-administered record or recall methodologies have the potential to reduce data entry expense and allow data collection for large numbers on multiple days over different time periods (27). They could therefore be more cost-effective and encourage countries for which cost has been a significant barrier to undertake surveys. For example, myfood24 is an online 24-hour dietary assessment tool that can be used for either of the EFSA-approved (16) 24hr recall or a food diary methods (27). It employs country-specific food databases and is currently in operation in Denmark, Germany and the United Kingdom. Technologies like this could reduce the onus on researchers by automatically coding food records (27). These benefits could encourage countries that historically lack national diet survey provision to undertake surveys and enable countries that already undertake surveys to implement these at more regular intervals. This would serve to increase the amount of dietary and nutrient intake data available in the WHO Europe remit, directly contributing to the WHO objective of strengthening and expanding nationally representative diet and nutrition surveys WHO (1).

### Energy and nutrient intakes

Energy and nutrient intake provision was documented from the *latest* survey collected after 2000 for each country for which we could locate intake data. For some countries, more recent surveys had been conducted (see Table 2), but intake data were not yet available in all cases. An additional limitation on data availability was the range of nutrients each survey covered. Of the countries that specified nutrient intakes, Germany and Belgium were the most likely to have gaps in reported intakes of macro- and micronutrients, respectively, and the Spanish ANIBES survey (28–30) only reported macronutrient data (see Appendix 3). This suggests that the reporting of nutrient intakes is inconsistent, making it harder to assess nutrient coverage and make inter-country comparisons.

Inconsistent age groupings across countries also make inter-country comparisons potentially problematic. In Andorra, the youngest age group spanned adults and children, meaning that although children were sampled, intake levels would not be valid in any comparisons. Future investigation could be undertaken using raw data and consistent age groups to obtain more reliable conclusions.

Differences in dietary methodologies may be a limiting factor when making inter-country comparisons. The relatively low levels seen in Turkish adult and child energy intakes compared to other countries could potentially be explained by methodological differences. The Turkish survey used a single 24hr recall, whereas the Belgian, Danish, German, Hungarian, Dutch, Norwegian and Spanish surveys, whilst using different methodologies (see Table 2), all collected data on multiple days. Collection on a single day is more likely to result in error due to less control over day-to-day variation (31).

Lack of alignment and completeness of national food composition databases and classification systems is a further limitation. For example, some food composition databases may not be updated to account for reformulated products, which could introduce differences and potential error in the energy and nutrient content of foods and therefore population intakes as reported in NDS. Common approaches to food composition databases are set out in more detail in the EFCOVAL study (144). Energy and nutrient intake values will be reported and discussed in more detail in future publications (145).

### Strengths and limitations

The strength of the current review is that it presents a unique, current overview of dietary survey characteristics in all WHO Europe countries since 1990. The existence of newer studies such as Bel-Serrat et al. (146) illustrates the fluidity of the situation and the need for updated, comprehensive reviews. This review includes surveys covering both adults and children; therefore, it provides a full picture of the current state of dietary survey provision across the life course. It also discusses methodologies, enabling insights into common methods and paving the way for future exploration of best practice and policy recommendations.

However, the surveys employed different methodologies, both between surveys and within long-running surveys with multiple collection waves, potentially making the task of comparing countries problematic. Despite this, we feel that there is still a need to use the available information to make inter-country comparisons where possible. Another limitation of the review was that we were unable to establish contact with nutrition experts or government officials who may be working in nutrition in some of the 19 countries where no surveys were found, which were mainly Central and Eastern European countries. Therefore, we cannot ascertain that these countries do not have any relevant dietary surveys. We also cannot assure that there are no other nationally representative surveys in countries where we obtained survey information from contacts. However, it is likely that these contacts would be aware of other surveys in their countries; in the distributed questionnaire, contacts were asked for details of all surveys in their country.

## Conclusion

This review found that less than two-thirds of the 53 countries in the WHO European region conducted national diet and nutrition surveys since 1990, with only 22 countries reporting nutrient intake data since 2000. The main survey gaps for both adults and children lie in the Central and Eastern European countries, where nutrition policies may lack an appropriate evidence base. Differing dietary assessment methodologies may have impact on the ability to make inter-country comparisons; existing efforts to harmonise NDS across all ages, particularly guidelines set by EFSA (16), should be encouraged, including beyond Western Europe. It would therefore be beneficial to target future efforts at standardising methodologies and filling knowledge gaps for the countries that have no surveys post-2000 in order to increase the information available for evidence-based policy planning. By establishing which countries have NDS, this review lays the foundation for a future review and stratified analyses of actual nutrient intakes across population groups in Europe.

## Appendix 1 Questionnaire concerning nationally representative diet and nutrition surveys and their methodologies

Please complete one questionnaire *per diet and nutrition survey (DNS)* for questions 1–3; if necessary make multiple copies. If there any questions in sections 1–3 that you cannot answer, please provide contact details of a person(s) who may be able to answer the outstanding questions.

Please email the completed questionnaire(s) to Holly Rippin fshr@leeds.ac.uk at the University of Leeds, who will be collating this information for the European Office of the World Health Organization.

**Country: xxx**

**Contact (please provide the correct contact person if this is incorrect): Prof/Dr. xxx**

1. **For each DNS carried out in your country since 1990 please fill in the below information:**
  Please note that any survey to be included should meet the following criteria:
  - The survey should collect dietary intakes across all food groups which are then converted into nutrient values.
  - The survey uses national population-based samples or representative regional samples.
  - The survey should not be restricted to a specific part of the population (e.g. children, occupational groups or patients).
  - Preferably there should be plans to repeat the survey later, unless it already has been repeated. You can also record standalone surveys.
  Survey name ………………………………………………
  Year(s) when survey data collected……………………....
  Dietary assessment method/tool used…………………...
  Genders included in sample………………………………
  Age ranges included in sample…………………………...
  Sample size (N)
  National or regional ……………………………..............
  Nationally representative (yes/no).………………........…
  Institute responsible for the survey………………………
  Key contact for survey………………...............…………
  Email for contact person listed above……………………
2. **Please provide details of any relevant publications e.g. summary reports, user guides (please provide web links)**
3. **Macro and micro nutrients included in your DNS (please tick all that apply):**
  Energy
  Total carbohydrates
  - Sugars
  - Sucrose
  - Starches
  - Fibre
  Total fat
  - Saturates
  - MUFA
  - PUFA
  - Trans fatty acids
  Protein
  Vitamins:
  - Folic acid
  - Niacin
  - Retinol equivalents
  - Riboflavin
  - Thiamine
  - B12
  - B6
  Minerals:
  - Calcium
  - Magnesium
  - Potassium
  - Sodium
  - Iron
  - Copper
  - Fluoride
  - Iodine
  - Selenium
  - Zinc

THANK YOU FOR TAKING THE TIME TO ANSWER THESE QUESTIONS.

## Appendix 2

**Table:** Macronutrient provision across dietary surveys

Country	Survey	Year	Energy (MJ and kcal)	Protein (g)	CHO g or %E	Sugars (g)	Sucrose (g)	Starches (g)	Fibre (g)	Total fat (g)	Saturates (g)	MUFA(g)	PUFA(g)	TFA(g)
Andorra	Evaluation of the nutritional status of the Andorran population	2004–2005	Y	Y	Y	Y			Y	Y	Y	Y	Y	
Austria	Austrian nutrition report	2010–2012	Y	Y	Y		Y		Y	Y	Y	Y	Y	
Belgium	The Belgian food consumption survey 2014–2015	2014–2015	Y	Y	Y	Y		Y	Y	Y	Y	Y	Y	Y
Bulgaria	National survey on nutrition of infants and children under 5 and family child rearing, 2007	2007	Y	Y	Y	Y			Y	Y	Y	Y	Y	
Cyprus	A study of the dietary intake of Cypriot children and adolescents aged 6–18 years	2009–2010	Y	Y	Y				Y	Y	Y	Y	Y	
Denmark	Danish dietary habits 2011–2013	2011–2013	Y	Y	Y		Y		Y	Y	Y	Y	Y	Y
Finland	The national FINDIET 2012 survey	2012	Y	Y	Y		Y		Y	Y	Y	Y	Y	Y
France	INCA2	2006–2007	Y	Y	Y	Y		Y	Y	Y	Y	Y	Y	
Germany	German national nutrition survey II	2005–2007	Y	Y	Y				Y	Y				
Hungary	Hungarian dietary survey 2009	2009	Y	Y	Y		Y		Y	Y	Y	Y	Y	
Iceland	The diet of Icelanders – a national dietary survey 2010–2011	2010–2011	Y	Y	Y	Y			Y	Y	Y	Y	Y	Y
Ireland	National pre-school nutrition survey	2010–2011	Y	Y	Y	Y		Y	Y	Y	Y	Y	Y	
Ireland	National children’s food survey	2003–2004	Y	Y	Y				Y	Y				
Ireland	National teens’ food survey	2005–2006	Y	Y	Y				Y	Y				
Ireland	National adult nutrition survey	2008–2010	Y	Y	Y	Y		Y	Y	Y	Y	Y	Y	Y
Italy	The third Italian national food consumption survey, INRAN-SCAI	2005–2006	Y	Y	Y	Y		Y	Y	Y	Y	Y	Y	
Latvia	Latvian national food consumption survey 2007–2009	2007–2009	Y	Y	Y				Y	Y	Y	Y	Y	
Lithuania	Study and evaluation of actual nutrition and nutrition habits of Lithuanian adult population	2013–2014	Y	Y	Y	Y			Y	Y	Y	Y	Y	
Netherlands	Dutch national food consumption survey (DNFCS) 2007–2010	2007–2010	Y	Y	Y	Y			Y	Y	Y	Y	Y	Y
Netherlands	Dutch national food consumption survey – young children (DNFCS 2008)	2005–2006	Y	Y	Y	Y			Y	Y	Y	Y		Y
Norway	Norkost3	2010–2011	Y	Y	Y		Y		Y	Y	Y	Y	Y	
Norway	Ungkost3	2015–2016	Y	Y	Y		Y		Y	Y	Y	Y	Y	
Portugal	National food and physical activity survey (IAN-AF)	2015–2016	Y	Y	Y	Y			Y	Y	Y	Y	Y	Y
Spain	ANIBES	2013	Y	Y	Y	Y			Y	Y	Y	Y	Y	
Spain	ENIDE 2011	2009–2010	Y	Y	Y				Y	Y	Y	Y	Y	
Sweden	Riksmaten 2010–2011 Swedish adult dietary survey	2010–2011	Y	Y	Y		Y		Y	Y	Y	Y	Y	
Turkey	Turkey nutrition and health survey 2010 (TNHS)	2010	Y	Y	Y				Y	Y	Y	Y	Y	
UK	National diet and nutrition survey (NDNS) Years 1–4	2008–2012	Y	Y	Y	Y		Y	Y	Y	Y	Y	Y	Y
**Total**	**28**		**28**	**28**	**28**	**14**	**7**	**6**	**28**	**28**	**25**	**25**	**24**	**9**

## Appendix 3

**Table:** Micronutrient provision across dietary surveys*

Country	Survey	Year	B9 (μg)	B3 (mg)	VA (μg)	B2 (mg)	B1 (mg)	B12 (μg)	B6 (mg)	VC (mg)	VD (μg)	VE (mg)	Ca (mg)	Mg (mg)	K (mg)	Na (mg)	Fe (mg)	Cu (mg)	I (μg)	Se (mg)	Zn (μg)
Andorra	Evaluation of the nutritional status of the Andorran population	2004–2005	Y	Y	Y	Y	Y	Y	Y	Y	Y	Y	Y	Y	Y	Y	Y				Y
Austria	Austrian nutrition report	2010–2012	Y	Y	Y	Y	Y	Y	Y	Y	Y	Y	Y	Y	Y	Y	Y		Y		Y
Belgium	The Belgian food consumption survey 2014–2015	2014–2015	Y			Y	Y	Y	Y	Y	Y		Y			Y	Y		Y		
Bulgaria	National survey on nutrition of infants and children under 5 and family child rearing, 2007	2007	Y	Y	Y	Y	Y	Y	Y	Y	Y	Y	Y	Y		Y	Y				Y
Cyprus	A study of the dietary intake of Cypriot children and adolescents aged 6–18 years	2009–2010			Y	Y	Y		Y	Y			Y	Y	Y	Y	Y				
Denmark	Danish dietary habits 2011–2013	2011–2013	Y	Y	Y	Y	Y	Y	Y	Y	Y	Y	Y	Y	Y	Y	Y		Y	Y	Y
Finland	The national FINDIET 2012 survey	2012	Y	Y	Y	Y	Y	Y	Y	Y	Y	Y	Y	Y	Y	Y	Y	Y	Y	Y	Y
France	INCA2	2006–2007	Y	Y	Y	Y	Y	Y	Y	Y	Y	Y	Y	Y	Y	Y	Y	Y	Y	Y	Y
Germany	German national nutrition survey II	2005–2007	Y	Y	Y	Y	Y	Y	Y	Y	Y	Y	Y	Y	Y	Y	Y		Y		Y
Hungary	Hungarian dietary survey 2009	2009	Y	Y	Y	Y	Y	Y	Y	Y	Y	Y	Y	Y	Y	Y	Y	Y			Y
Iceland	The diet of Icelanders – a national dietary survey 2010–2011	2010–2011	Y	Y	Y	Y	Y	Y	Y	Y	Y	Y	Y	Y	Y	Y	Y	Y	Y	Y	Y
Ireland	National pre-school nutrition survey	2010–2011	Y	Y	Y	Y	Y	Y	Y	Y	Y	Y	Y	Y			Y	Y			Y
Ireland	National children’s food survey	2003–2004	Y	Y	Y	Y	Y	Y	Y	Y	Y	Y	Y	Y			Y	Y			Y
Ireland	National teens’ food survey	2005–2006	Y	Y	Y	Y	Y	Y	Y	Y	Y	Y	Y	Y			Y	Y			Y
Ireland	National adult nutrition survey	2008–2010	Y	Y	Y	Y	Y	Y	Y	Y	Y	Y	Y	Y	Y	Y	Y	Y			Y
Italy	The third Italian national food consumption survey, INRAN-SCAI	2005–2006			Y	Y	Y	Y	Y	Y	Y	Y	Y	Y	Y		Y				Y
Latvia	Latvian national food consumption survey 2007–2009	2007–2009	Y	Y	Y	Y	Y	Y	Y	Y	Y	Y	Y	Y	Y	Y	Y	Y	Y		Y
Lithuania	Study and evaluation of actual nutrition and nutrition habits of Lithuanian adult population	2013–2014	Y	Y	Y	Y	Y	Y	Y	Y	Y	Y	Y	Y	Y	Y	Y	Y	Y	Y	Y
Netherlands	Dutch national food consumption survey (DNFCS) 2007–2010	2007–2010	Y		Y	Y	Y	Y	Y	Y	Y	Y	Y	Y	Y	Y	Y	Y	Y	Y	Y
Netherlands	Dutch national food consumption survey – young children (DNFCS 2008)	2005–2006	Y		Y	Y	Y	Y	Y	Y	Y	Y	Y	Y			Y	Y		Y	
Norway	Norkost3	2010–2011	Y		Y	Y	Y	Y	Y	Y	Y	Y	Y	Y	Y	Y	Y				
Norway	Ungkost3	2015–2016	Y		Y	Y	Y	Y	Y	Y	Y	Y	Y	Y	Y	Y	Y			Y	
Portugal	National food and physical activity survey (IAN-AF)	2015–2016	Y	Y	Y	Y	Y	Y	Y	Y	Y	Y	Y	Y	Y	Y	Y				Y
Spain	ANIBES	2013																			
Spain	ENIDE 2011	2009–2010	Y	Y	Y	Y	Y	Y	Y	Y	Y	Y	Y	Y	Y	Y	Y		Y	Y	Y
Sweden	Riksmaten 2010–2011 Swedish adult dietary survey	2010–2011	Y	Y	Y	Y	Y	Y	Y	Y	Y	Y	Y	Y	Y	Y	Y			Y	Y
Turkey	Turkey nutrition and health survey 2010 (TNHS)	2010	Y	Y	Y	Y	Y	Y	Y	Y	Y	Y	Y	Y	Y	Y	Y		Y		Y
UK	National diet and nutrition survey (NDNS) Years 1–4	2008–2012	Y	Y	Y	Y	Y	Y	Y	Y	Y	Y	Y	Y	Y	Y	Y	Y	Y	Y	Y
**TOTAL**	**28**		**25**	**20**	**26**	**27**	**27**	**26**	**27**	**27**	**26**	**25**	**27**	**26**	**21**	**22**	**27**	**13**	**13**	**11**	**22**

* Key

**Table:**

B9	Folic acid
B3	Niacin
VA	Vitamin A (retinol equivalent)
B2	Riboflavin
B1	Thiamine
B12	Vitamin B12
B6	Vitamin B6
VC	Vitamin C
VD	Vitamin D
VE	Vitamin E
Ca	Calcium
Mg	Magnesium
K	Potassium
Na	Sodium
Fe	Iron
Cu	Copper
I	Iodine
Se	Selenium
Zn	Zinc
	
